# Comparison of subclavian vein and inferior vena cava collapsibility index in the intensive care unit

**DOI:** 10.1590/1806-9282.20240786

**Published:** 2024-12-02

**Authors:** Serkan Solak, Suheyla Karadag Erkoç, Onat Ahmet Bermede, Mustafa Kemal Bayar

**Affiliations:** 1 Ankara University, Faculty of Medicine, Department of Anesthesiology and Reanimation – Ankara, Turkey.

**Keywords:** Intensive care units, Subclavian vein, Ultrasonography, Vena cava, inferior

## Abstract

**OBJECTIVE:**

The aim of this study was to evaluate the correlation between changes in the inferior vena cava collapsibility index and subclavian vein collapsibility index in patients undergoing passive leg raising tests in the intensive care unit, considering that respiratory changes affecting the inferior vena cava may similarly affect the subclavian vein.

**METHODS:**

This single-center observational study was conducted on patients aged between 18 and 85 years who underwent passive leg raising in the intensive care unit. When the patient was 45° above the bed, the inferior vena cava and subclavian vein were displayed using ultrasonography; subclavian vein collapsibility index and inferior vena cava collapsibility index values were calculated. After the initial values were recorded, passive leg raising was performed, and the initial measurements were repeated. The CI values measured after passive leg raising were subtracted from those calculated before passive leg raising to determine the changes (Δ) in inferior vena cava and subclavian vein collapsibility indices.

**RESULTS:**

The study was conducted with a total of 64 patients. The mean±standard deviation values for ΔIVC-CI% and ΔSCV-CI% variables were found as 8.97±8.89 and 10.31±10.81, respectively. There were no statistically significant differences in values of ΔIVC-CI% and ΔSCV-CI% (p=0.297). In the Bland-Altman plot, because there were only two values exceeding the +1.96 SD and -1.96 SD limits, it can be said that the agreement between ΔIVC-CI% and ΔSCV-CI% was adequate.

**CONCLUSION:**

ΔSCV-CI% values are compatible and correlated with ΔIVC-CI% values. Inferior vena cava and subclavian vein responded similarly to fluid changes during passive leg raising.

## INTRODUCTION

Fluid resuscitation is the first-line treatment to increase cardiac output and provide tissues with better oxygenation in critically ill patients^1^. However, excessive fluid may disrupt oxygen transfer. Additionally, it may cause pulmonary and peripheral edema and abdominal compartment syndrome, which increase mortality significantly^2^. Therefore, assessment of fluid responsiveness is central in critically ill patients^3^.

Ideally, methods for assessing fluid responsiveness should be noninvasive, accurate, reliable, and sustainable. Recently, there has been a growing interest in bedside ultrasonography (USG) to evaluate the volume status of a patient, such as measurement of the inferior vena cava (IVC) distensibility and collapsibility index. IVC assessment is a dynamic parameter in which a transpulmonary pressure change is transmitted to the right heart via heart–lung interactions. Subsequently, these changes influence venous return during the respiratory cycle. Therefore, respiratory activity affects the diameter of the IVC^4,5^.

Imaging errors or any factors that affect IVC compliance may hinder accurate interpretations of IVC assessment. When abdominal USG is inadequate for imaging the IVC, assessment of the subclavian vein (SCV), which is similarly influenced by respiration-related variables and has an extrathoracic location, may be used as an alternative method to predict fluid responsiveness. Furthermore, the fact that the SCV is not affected by increases in intraabdominal pressure due to its anatomic location may have an advantage over the IVC. It is expected that the SCV could provide more reliable results because it is located in a more sheltered anatomic position against compression induced by interventions with USG probes (as one end of the ultrasound probe will be over the clavicula, this will prevent compression on the SCV) in comparison with the internal jugular vein and femoral vein^6,7^.

The hypothesis of this study was formed by expecting that the respiratory changes affecting the IVC would similarly affect the SCV and related collapsibility measurements. In this study, we investigated the correlation between the inferior vena cava collapsibility index (IVC-CI) and the subclavian vein collapsibility index (SCV-CI) in patients undergoing passive leg raise (PLR) maneuvers. The correlation between IVC-CI and SCV-CI in patients under invasive mechanical ventilation (IMV) and spontaneous breathing was also evaluated.

## METHODS

After ethics committee approval was obtained, this single-center, observational study was conducted among patients whose fluid status was evaluated through noninvasive techniques admitted to the intensive care unit (ICU) between December 2018 and December 2019.

### Patients

All patients aged between 18 and 85 years undergoing PLR tests were included in the study. The study included participants who provided informed consent after the informed consent form was explained to the patient and/or their relatives.

Exclusion criteria were body mass index (BMI) >35 kg/m^2^, previous laparotomy, previously diagnosed increased intraabdominal pressure (>12 mm/Hg), IVC or SCV that could not be visualized with USG, arrhythmia, acute respiratory distress syndrome (ARDS), a mass in the proximal one-third clavicula, thoracic venous outlet syndrome, head trauma, and impaired lower extremity movement due to multiple traumas.

Demographic data, including age, sex, comorbid diseases, diagnosis, postoperative hospitalization data, the unit of admission, and Acute Physiology and Chronic Health Evaluation (APACHE II) scores, were collected. In addition, heart rate, invasive or noninvasive blood pressure, peripheral oxygen saturation of patients, and the ventilation modes of those under invasive mechanical ventilator support were recorded.

### Ultrasonographic examination

The USG measurements were performed by a single individual with sufficient experience in this field. The head of the bed was elevated to 45°, and no further changes were made in the mechanical ventilator settings during the measurement period. Patients were assessed in terms of venous thoracic outlet syndrome by using the dynamic method of abducting the arms to 90° through hyper abduction before measurement of the SCV.

The maximum and minimum diameters were measured, and the SCV-CI and IVC-CI basal values were calculated by imaging the SCV and IVC using USG (Fujifilm Sonosite FC1, Inc., Bothell, WA 98021 USA). Subsequently, the patient was placed supine, and PLR was performed as described elsewhere^8^. Heart rates, invasive or noninvasive blood pressures, oxygen saturation levels, SCV, and IVC maximum and minimum diameters were recorded within 3 min in this position.

The hepatic vein–IVC junction and IVC–right atrium junction were visualized in the subcostal window using a 1–5 MHz sector probe for IVC-CI calculation. The minimum and maximum diameters of the IVC were measured using M-mode 2 cm from the right atrium and distal to the hepatic vein.

IVC-CI was calculated based on the following formula:


$$\text{IVC}-\text{CI}=\left({\text{D}}_{\text{max}}\;-{\text{D}}_{\text{min}}\right)\;/{\text{D}}_{\text{max}}\text{x1}00.$$

For SCV-CI calculation, from the proximal one-third level of the clavicula where the upper tip of the probe would be over the clavicula (for not compressing the vein), SCV maximum and minimum diameters were measured in the M-mode on the long axis using a 6–15 MHz linear probe.

SCV-CI was calculated using the following formula:


$$\text{SCV}-\text{CI}=\left({\text{D}}_{\text{max}}\;-{\text{D}}_{\text{min}}\right)\;/{\text{D}}_{\text{max}}\text{x 1}00$$

The difference in CI calculated before and after PLR is denoted as “Δ.” It was calculated using the following formula:


$$\begin{array}{l} \Delta \text{IVC}-\text{CI}\%=\text{Pre}-\text{PLR IVC}-\text{CI}\%-\text{Post}-\text{PLR IVC}-\text{CI}\%\;\\ \Delta \text{SCV}-\text{CI}\%=\text{Pre}-\text{PLR SCV}-\text{CI}\%-\text{Post}-\text{PLR SCV}-\text{CI}\% \end{array}$$

### Statistical analysis

An a priori power analysis was conducted using the G*Power3 software (Faul, Erdfelder, Lang, & Buchner, 2007) to test the difference between two independent group means. The results showed that a sample size of 54 patients would be sufficient to detect a correlation coefficient of 0.80 with a significance level of 0.05 and a power of 80% when the sample size was calculated over five patients with a correlation test.

Descriptive statistics are reported as mean±standard deviation and median (minimum–maximum) for quantitative variables and patient numbers (frequency) and percentages for qualitative variables. Student’s t-test was used when there was a normal distribution, and the Mann-Whitney U test was used when there was a non-normal distribution. Qualitative variables with more than two categories were assessed using one-way analysis of variance (ANOVA) when normal distribution was provided, and the Kruskal-Wallis test was used as a nonparametric test. The Bland-Altman method was used to check the agreement between two variables. Pearson’s correlation analysis was used for normally distributed data, and Spearman’s correlation analysis was used for non-normally distributed data. For determining relationships/differences between two quantitative-dependent variables, a paired-sample t-test was used for normally distributed data, and the Wilcoxon test was used for the non-normally distributed data. The statistical significance level was taken as 0.05 (two-tailed).

## RESULTS

A total of 64 patients were included. Among the patients, 39 (60.9%) were male, and 25 (39.1%) were female, with a mean age of 61.08±14.66 years. The demographic data of the patients, their APACHE II scores, diagnosis at the time of ICU admission, and ventilation status are presented in Table 1. In total, 28 patients were followed up with spontaneous respiration without invasive or non-IMV, 25 patients were under controlled IMV, and 11 patients were under spontaneous IMV (Table 1).

**Table 1:** Demographic data, Acute Physiology and Chronic Health Evaluation II scores, and respiratory status.

Age, y, mean±SD	61.08±14.66
Sex (M/F)	39/25
Body mass index, kg/m^2^, mean±SD	25.24±4.08
APACHE II scores	17.31±10.02
Diagnosis of hospitalization, n (%)	
Sepsis	11 (17.2)
Postoperative	27 (42.1)
Cardiac arrest	3 (4.7)
Intoxication	2 (3.1)
Respiratory failure	19 (29.7)
Intravehicular traffic accident	1 (1.6)
Diabetic ketoacidosis	1 (1.6)
Respiratory status, n (%)	
IMV-control mode	25 (39)
IMV-CPAP mode	11 (17.2)
Spontaneous respiration	28 (43.8)

CPAP: continuous pressure airway ventilation; SD: standard deviation; APACHE II: Acute Physiology and Chronic Health Evaluation II; IMV: invasive mechanical ventilation.

**Figure 1:**
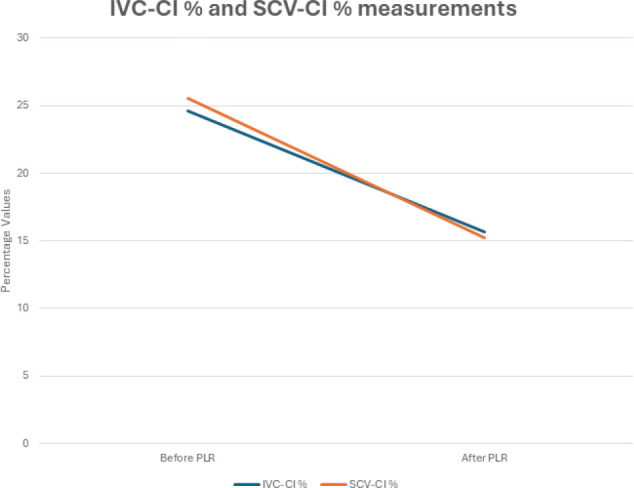
Graph of ΔIVC-CI % and ΔSCV-CI % variables.

The mean, standard deviation, and median values for the ΔIVC-CI% variable were found as 8.97±8.89 [median: 8.00 (-7.00)–(28.00)]. For the ΔSCV-CI variable, the mean, standard deviation, and median values were 10.31±10.81 [median: 9.00 (-9.00)–(39.00)]. There were no statistically significant differences in the ΔIVC-CI% and ΔSCV-CI% changes (p=0.297, paired-sample t-test). As seen in the graph, ΔIVC-CI and ΔSCV-CI progressed in parallel to each other (Figure 1).

The IVC-CI and SCV-CI measurements determined before and after the PLR tests were correlated. Only two values were outside the limits of +1.96 SD and -1.96 SD for the data obtained in the Bland-Altman plot analysis. The agreement between these two differences was adequate.

A strong and statistically significant relationship between the before and after PLR IVC-CI and SCV-CI measurements was detected (respectively, R^2^=0.554 and R^2^=0.408) (p<0.001).

The correlation between the IVC-CI and SCV-CI measurements before and after the PLR tests in the patients who were under controlled mechanical ventilation support was determined to be higher than that in the patients who were under spontaneous mechanical ventilation or spontaneous respiration (Table 2).

**Table 2:** Relationship between inferior vena cava collapsibility index and subclavian vein collapsibility index before and after passive leg raising based on the respiratory status.

	Values before PLR		Values after PLR	
	Correlation coefficient	p-value	Correlation coefficient	p-value
Patient under controlled IMV (n=25)	0.863	<0.001^a^	0.766	<0.001^a^
Patients with spontaneous respiration (n=28)	0.615	<0.001^a^	0.503	0.006^a^

^a^Pearson correlation coefficient. PLR: passive leg raising; IMV: invasive mechanical ventilation.

## DISCUSSION

This study observed that ΔSCV-CI and ΔIVC-CI data were consistent with each other. This result indicates that SCV and IVC responded similarly to internal fluid changes associated with PLR. Additionally, we found a strong correlation between SCV-CI measured before PLR and IVC-CI measured before PLR, and this correlation also remained similar in post-PLR measurements. This correlation was higher in patients under controlled IMV support than in those under spontaneous respiration without MV support.

During the inspiratory phase of positive pressure mechanical ventilation in patients without breathing effort, venous return decreases, and the IVC diameter increases. However, venous return increases, and the IVC diameter decreases in patients breathing spontaneously. Fluid responsiveness can be predicted by evaluating IVC collapsibility and distensibility indices, which can be calculated by using the changes measured in the diameter of IVC in response to spontaneous respiration or mechanical ventilation (minimum and maximum diameters)^9^. In a study including 23 patients with septic shock and under controlled mechanical ventilation, Barbier et al.^10^ concluded that IVC-DI was a good marker in determining intravascular fluid status. When the IVC-DI is ≥18%, patients will benefit from fluid resuscitation with 90% sensitivity and 90% specificity. In another study where the effectiveness of IVC-CI was examined in patients with acute circulatory failure with spontaneous respiration, it was concluded that the IVC-CI predicted fluid responsiveness with 70% sensitivity and 80% specificity when the collapsibility index was ≥40%^11^. In general, it has been reported that respiration-dependent changes in IVC diameter provide reliable results in assessing fluid status^12,13^. Although calculating IVC collapsibility and distensibility indices is easy to apply, noninvasive, and reproducible for determining fluid status, IVC imaging may not be feasible due to obesity, abdominal surgery, and intestinal gas. Moreover, increased abdominal pressure may lead to misleading results in IVC measurements dependent on respiratory changes^14^. When evaluating our results, the correlation between SCV-CI and IVC-CI values suggests the potential usability of SCV-CI in assessing intravascular fluid status.

In a study involving 21 patients evaluating the usability of SCV-CI and the SCV variability index, Giraud et al.^15^ examined the changes in cardiac flow following 500 mL 0.9% NaCl fluid bolus, changes in SCV-CI and the SCV variability index using transpulmonary thermodilution and pulse contour analysis (PiCCO™) in patients with controlled mechanical ventilation under deep sedation. They observed a correlation between the SCV variability index and SCV collapsibility index with ΔCO. In our study, the relationship between the SCV and IVC and Δ values related to internal fluid changes were analyzed in both spontaneously breathing and mechanically ventilated patients. We observed that SCV-CI and IVC-CI values were correlated between patients receiving ventilation support or spontaneously breathing and related patients.

Peachey et al.^16^ examined IVC-CI, stroke volume, and cardiac output before and after PLR in 33 volunteers following 8 h of fasting. A significant increase in cardiac output (mean: 22.3%) and a substantial decrease in IVC-CI were determined (mean: -29.2%) after PLR. Likewise, we observed significant reductions in IVC-CI and SCV-CI after PLR.

This study has several limitations. First, the reliability of USG measurements requires expertise, and measurements can vary depending on the individual. Second, we did not include a cardiac flow measurement component to assess the effectiveness of PLR. Therefore, we cannot discuss the sensitivity of the SCV in fluid responsiveness.

In conclusion, there was a significant correlation between the IVC and SCV collapsibility indices in patients undergoing PLR tests. This correlation was higher in patients under controlled IMV. Further studies need to be conducted, including broader patient groups where cardiac flow rate measurements are made after PLR tests to evaluate the results’ clinical significance.
